# Biosynthesis of selenium nanoparticles by *Aloe vera* leaf extract and its biomedical applications

**DOI:** 10.1186/s11671-026-04489-7

**Published:** 2026-03-30

**Authors:** Aya M. El-Ebidy, Amr M. Mowafy, Heba M. M. Abdel-Aziz

**Affiliations:** 1 Botany Department, Faculty of Science, Mansoura University, Mansoura, Egypt; 2 Department of Biological Sciences, Faculty of Science, New Mansoura University, New Mansoura City, Egypt

**Keywords:** *Aloe vera*, Anti-inflammatory, Anticancer, Caco2 cell lines, DPPH scavenging, Selenium nanoparticles

## Abstract

**Background:**

*Aloe vera* has been cultivated mainly for medicinal purposes, with over 75 biologically active compounds in its gel. Selenium nanoparticles (SeNPs) are novel selenium sources characterized by great biocompatibility, low toxicity, stability, selectivity, and extensive uses. This study emphasizes the significant role of phytofabricated SeNPs in several biomedical applications.

**Methods & results:**

SeNPs were synthesized utilizing Aloe vera leaf extract. The biosynthesized SeNPs were characterized using UV–Vis, EDX, XRD, zeta potential, FTIR, and TEM analyses. The antioxidant, antibacterial, anti-inflammatory, and anticancer effects, along with the impact on antioxidant enzymes of biosynthesized SeNPs, were evaluated. The biomedical functions of SeNPs were concentration-dependent, showing antibacterial activity against both Gram-positive bacteria (*Bacillus cereus*, *Staphylococcus aureus*, and *Bacillus subtilis*) and Gram-negative (*Escherichia coli*, *Salmonella typhimurium*, and *Klebsiella pneumonia*). Additionally, SeNPs exhibited antioxidant activity, with DPPH scavenging from 77.39 ± 0.895% at 120 µg mL^–1^ to 20.75 ± 0.952% at 5 µg mL^–1^. SeNPs showed anti-inflammatory activity at 31.25 µg mL^–1^, inhibiting of bovine serum albumin (BSA) denaturation by 79.87 ± 1.21%, compared to 82.23 ± 1.127% for diclofenac sodium. SeNPs exhibited anticancer efficiency against MCF7 and Caco2 cells with IC50 values of 312.02 ± 3.25 and 120.96 ± 1.87 µg mL^− 1^, respectively, and were biocompatible with the normal WI38 cells. Moreover, there was a notable decline in the enzyme activities CAT activity decreased by 48.32% and 59.39% and SOD by 43.26% and 54.52% in the Caco2 and MCF-7 cell lines, respectively.

**Conclusions:**

The Bio-fabricated SeNPs exhibited nontoxicity and eco-friendliness, demonstrating notable antimicrobial efficacy, particularly against *S. aureus*, *K. pneumonia*, and *B. subtilis*. They also possess high DPPH scavenging capabilities, anti-inflammatory properties, and exhibit superior anticancer activity on the Caco2 cancerous cell line compared to the MCF7 cell line, while retaining WI38 normal cells, underscoring their potential in medical applications.

**Graphical abstract:**

**Supplementary Information:**

The online version contains supplementary material available at 10.1186/s11671-026-04489-7.

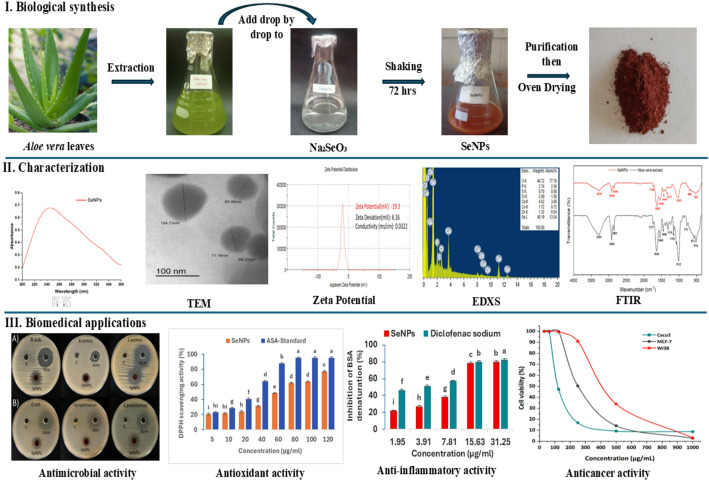

## Introduction

Nanomaterials (1–100 nm) have unique physical and chemical characteristics that differentiate them from bulk materials [1, 2]. Currently, nanotechnology is one of the most demanding research disciplines that has attracted substantial interest from chemists, biologists, physicists, and engineers for various applications in emerging technologies and consumer products. These applications such as in biomedicine, renewable energy, agriculture, antibacterial purposes, optical, sensors, catalytic devices, electronic appliances, and other products used for personal care, water, and soil treatment [3].

Nanoparticles are synthesized by various processes, including physical, chemical, and biological mechanisms. Physical and Chemical procedures are generally undesirable due to complicated synthesis conditions, generation of hazardous byproducts, biosafety issues, and increased costs [1]. Among the several techniques developed to produce nanoparticles (NPs) with specific sizes and shapes, as well as distinctive properties in diverse solvents, biogenic approaches are gaining significant attention [4, 5].

Metal nanoparticles have received significant attention for their potential as antibacterial agents [6]. As an important component of selenoenzymes, selenium (Se) is a vital micronutrient that is essential for a variety of basic biological functions in the human body and in the majority of living organisms [7, 8]. According to Hariharan et al. [9], selenium in different nanoforms has demonstrated more antioxidant and anticancer potential and less toxicity than organic and inorganic forms. Furthermore, the bioavailability, minimal toxicity, and elevated biological activity of SeNPs provide them as suitable choices for substituting selenium in medicines or nutritional supplements [10]. Selenium nanoparticles (SeNPs) were utilized as an antioxidant, chemopreventive, antibacterial, antifungal, and therapeutic agent and photocatalyst [11, 12].

The biosynthesis of SeNPs, a beneficial and economical process, is related to the removal of selenite and selenate from effluents. Consequently, there is now greater interest in the biological synthesis of SeNPs [13]. The nanoparticles production using a variety of plants and extracts from them may be beneficial than other biological synthetic techniques, requiring highly complicated microbial culture maintenance procedures [14].

Plant extract-mediated nanoparticles enhance SeNPs synthesis by providing reducing and stabilizing agents, as well as herbal capping. Moreover, bioactive constituents like tannins, quinine, steroids, carbohydrates, glycosides, saponins, phenols, flavonoids, alkaloids, and proteins present in plant extracts can accelerate the synthesis of SeNPs in a singular step [15, 16]. Consequently, the incorporation of phytochemicals into nanoparticles may give them supplementary functional characteristics [17].

*Aloe vera* has been known for its numerous therapeutic benefits for thousands of years. It involves valuable nutritional and antipathogenic components [14]. *Aloe vera* contains numerous natural bioactive compounds that are both complex and simple. These include foaming agents, anthraquinones, aqueous polysaccharides, plant-derived alcohol, oleic acid, glycosides, and pyrocatechol. *Aloe vera* leaf demonstrates enhanced antibacterial efficacy in both alcoholic and water-soluble forms [18, 19]. SeNPs are less toxic form of selenium. FRAP, ABTS and DPPH assay results sequester that SeNPs prepared using *Aloe vera* extract possess more activity than extract alone [20].

This study aimed to utilize *Aloe vera* mediated SeNPs biosynthesis in an eco-friendly, economical, and simple approach. Firstly, a prepared plant extract was examined for its efficiency in the biological synthesis of SeNPs. Subsequently, the SeNPs were characterized using UV–vis, TEM, FT-IR, zeta potential, XRD, and EDX analysis. Antibacterial, antioxidant, anti-inflammatory, anticancer, and biocompatibility studies were conducted to examine the potential biological applications of biosynthesized SeNPs. This study discusses prospective applications of SeNPs in essential medical fields.

## Materials and methods

### *Aloe vera* leaf extract preparation

*Aloe vera* plants were obtained from the Botanical Garden in Faculty of Agriculture, Mansoura University, Mansoura, Egypt (latitude ~ 31.04° N, longitude ~ 31.38° E). Voucher number: 270 in the Botanical Herbarium, Botany Department, Faculty of Science, Mansoura University, Mansoura, Egypt. At room temperature (25 °C ± 2), a 30-gram portion of freshly cleaned *Aloe vera* leaf were dissolved in 20 ml of sterilized deionized water in a blender for 10 min. to form 45 ml leaf extract which was filtered using Whatman filter paper no. 1 and stored at 4 °C for further study. The pH of the obtained extract was 5.

### Biosynthesis of SeNPs

The biosynthesis of SeNPs occurred following a modified protocol provided by Vyas and Rana [20]. A flask containing 25 ml of Na_2_SeO_3_ (5 mM) solution was placed on a magnetic stirrer. *Aloe vera* leaf extract (45 ml) was added drop by drop to Na_2_SeO_3_ while stirring with a ratio of 9:5. After 72 h in a shaker under dark conditions the color of Na_2_SeO_3_ change was seen from pale yellow to brick red. Unreacted reagents were subsequently removed from the reaction mixture by centrifuging it for 20 min. at 12,000 rpm at 25 °C. The pellet containing synthesized nanoparticles were rinsed with ethanol, followed by deionized water, and then subjected to centrifugation. The precipitates that resulted were dried at 60 °C for 24 h and subsequently ground into a powder and kept for further study.

### Characterization of SeNPs

Following 72 h of incubation, the SeNPs’ synthesis was initially verified by a color shift from pale yellow to brick red. Biosynthesized SeNPs were examined using a variety of analytical methods to determine their physical and chemical properties.

#### UV–visible spectroscopic analysis of SeNPs

The spectral scan of the obtained SeNPs was performed by 6705 UV-VIS spectrophotometer (Jenway, England) from 200 to 800 nm, utilizing deionized water as a reference.

#### Detection of SeNPs by transmission electron microscopy (TEM)

Further characterization of the obtained SeNPs has been done using TEM to determine morphology and size. TEM was carried out by dispersing a minimal quantity of material in water, sufficient to create a little turbid solution. An ultrasonicator was used to homogenize the solution to ensure that the particles were distributed uniformly. Pipetting a drop of the solution was employed to apply it to carbon-coated grids. After drying, the grid was fixed in the specimen holder for observation. In Mansoura, Egypt, at the Electron Microscope Unit of Mansoura University, a TEM examination was achieved using a JEOL JEM 2100 (Tokyo, Japan).

#### Zeta- potential of the synthesized SeNPs

Using a Nano-zs90 zeta analyzer (Malvern, UK), the zeta-potential of the synthesizes SeNPs was determined.

#### EDXS (energy-dispersive X-ray spectroscopy)

Using a JEOL JSM 6510 lv SEM (JEOL Ltd., Japan), the elemental composition of the substance was determined via the EDXS study in Mansoura, Egypt, at the Electron Microscope Unit of Mansoura University.

#### Fourier transform infrared (FTIR) spectroscopy analysis

FTIR examined the surface chemistry of the obtained nanoparticles by identifying functional groups via infrared absorption frequencies ranging from 400 to 4000 cm^− 1^. SeNPs were dispersed in a dry KBr matrix, formed into a SeNPs-KBr disk, and examined by a Nicolet iS-10 FTIR spectrometer (Thermo Scientific, USA).

#### X-ray diffraction (XRD) analysis

Using a CuKα radiation source (λ = 1.5406 A°, applied voltage 10 kV, current 30 mA), the crystallinity and structural characteristics of the SeNPs were determined in a Bruker D2 Phaser 2ⁿᵈ Gen diffractometer, Germany. At a scanning rate of 2°/min, data was collected for 2θ values between 0 and 70.

### Biological applications of SeNPs

#### Antimicrobial activity of SeNPs

Agar well diffusion method was employed to evaluate the antibacterial efficiency of SeNPs against six microbiological strains, containing both Gram-negative (*Escherichia coli* ATCC 10536, *Klebsiella pneumonia* ATCC 10031, and *Salmonella typhimurium* ATCC 25566*)*, and Gram-positive bacteria *(Bacillus cereus* EMCC number 1080, *Bacillus subtilis* DMS 1088, and *Staphylococcus aureus* ATCC 6538). A spectrophotometer set to 600 nm was used to adjust the turbidity of the bacterial inoculum to the 0.5 McFarland standard. The plate was inoculated by distributing a volume of the bacterial inoculum on the entire surface of the agar. A 9 mm diameter opening was aseptically created using a sterilized cork borer or tip. A volume of 100 µL of SeNPs (15 mg mL^− 1^), standard antibiotic (azithromycin 2 mg mL^− 1^), and DMSO (negative control) were inserted into the generated wells separately. The plates were then incubated at 37 °C for 24 h after being refrigerated for 2 h (*n* = 3). The formation of clear zones was monitored around the wells [21].

#### Antioxidant activity using DPPH assay

The DPPH colorimetric assay was applied for determining the antioxidant activity of SeNPs. The DPPH solution was utilized as the negative control and ascorbic acid as the positive control, in accordance with the assay described by Kitts et al. [22]. In this experiment, different concentrations of biosynthesized SeNPs (5–120 µg mL^–1^) were prepared in deionized water. Subsequently, one mL of SeNPs solution was placed into a test tube containing one mL of DPPH (dissolved in methanol) at a concentration of 0.1 mM. Immediately samples were stored subsequent to the addition of the DPPH• solution at room temperature in dark conditions for 30 min. Next, the absorbance of each sample was measured at 517 nm (*n* = 3). Also, IC50 was calculated, the IC50 (inhibitory concentration for 50% of cellular viability) was also calculated.

The DPPH scavenging activity of the samples was determined using Eq.

$${\text{DPPH scavenging activity}} (\% ) = [ (\mathrm{A} - {\mathrm{B}}) / \mathrm{A}]  \times 100 $$

Where A is the absorbance of the DPPH and B is the absorbance of SeNPs.

#### Anti-inflammatory activity of SeNPs

SeNPs were evaluated for their anti-inflammatory capabilities by preventing protein denaturation, in accordance with previously published studies with little modification. Initially, we took 0.45 mL of 1% bovine serum albumin (BSA) dissolved in phosphate buffer at pH 6.4, followed by adding 0.05 mL of SeNPs at varying concentrations. Following a 20-minute incubation at 37 °C, the mixtures were further heated to 80 °C for 8 min. Spectrophotometric measurements were made at 660 nm after the samples had cooled down (*n* = 3). A typical pharmaceutical agent, diclofenac sodium, was utilized as a positive control, the BSA solution served as the negative control [23].

$${\mathrm{Inhibition}}(\% ){\text{ = }}({\text{Control OD value - Test sample OD value}}){\mathrm{/}}({\text{Control OD value}}) \times 100 $$

#### Cytotoxicity assay of SeNPs

The SeNPs cytotoxicity was estimated in vitro on the cell line of human normal lung fibroblast (WI-38) and two types of cancer cells (colon Caco2 and breast MCF7), sourced from the American Type Culture Collection. The cytotoxicity of SeNPs was estimated utilizing the MTT assay (3-(4,5-dimethyl thiazol-2-yl)-2,5-diphenyltetrazolium bromide) [24]. In each well of 96-well plates, 1 × 10^5^ cells/ml (100 µl/well) were injected and incubated for 24 h at 37 °C to establish a full monolayer sheet. The cell monolayer was rinsed twice with wash media after the growth medium was removed.

Multiple concentrations of SeNPs were prepared in RPMI medium and using 2% serum as the maintenance medium. Three wells served as the control and received only maintenance medium, while 0.1 ml of every concentration was tested in separate wells. At 37 °C Incubation of the plate was conducted for 24 h. MTT was reduced by preparing a yellow MTT solution (5 mg/ml in PBS) and 20 µl of the solution was added to each well. Subject to a shaking table for 5 min at 150 rpm make sure that the MTT is fully integrated into the media, then incubate at 37 °C for 4 h with 5% CO_2_ to facilitate MTT metabolism. Following the combination of purple formazan (MTT metabolic product) with 200 µl of DMSO, put it on a shaking table for 5 min at 150 rpm; Following that, A plate reader from the United States, the EXL 800, was used for determining the absorbance at 570 nm (*n* = 3).

The formula below has been used to calculate the cell viability percentage:

$${\mathrm{Viability}} \% = (\text{Test OD}/\text{Control OD})  \times {\mathrm{1}}00~~~~~{\text{Cytotoxicity }}\% = {\mathrm{1}}00 - {\mathrm{Viability}}\% $$

#### Determination of antioxidant markers

The two cancer cell lines, colon Caco2 and breast MCF7, were subjected to IC_50_ concentrations of SeNPs treatment for 24 h, as determined by the MTT experiment. Subsequently, the cells were harvested, lysed RIPA lysis reagent for 30 min on ice, and centrifuged at 12,000 g for 1 min at 4 °C and the obtained cell lysate was used for the enzyme assays.

Catalase (CAT) activity was evaluated with a catalase measurement kit (Biodignostics Co.) in accordance with the manufacturer’s guidelines. To the assay mixture of 50 ul of Cell lysates were treated with 50 ul H_2_O_2_ 500 μm and 500 ul phosphate buffer pH 7.0 for 3 min the remaining H_2_O_2_ was reacted with a substrate to yield N-4-antipyryl-3-chloro-5-sulfonate-p-benzoquinonemonoimine, exhibiting a maximum absorbance at 540 nm (*n* = 3). Catalase activity was estimated by measuring the breakdown of H_2_O_2_ using a spectrophotometer (Biosystem 310 plus). The Bradford method was employed to quantify protein concentration [25].

Superoxide dismutase (SOD) activity was measured by determining the nitroblue tetrazolium (NBT) photoreduction inhibition by the SOD enzyme. The reaction mixture comprised 50 mM sodium phosphate buffer (pH 7.6), 10 µM riboflavin, 50 µM NBT, 0.1 mM EDTA, 50 mM sodium carbonate, 12 mM L-methionine, and 100 µL crude extract, culminating in a total volume of 3 mL. The assay started by subjecting the reaction mixture to white light for 15 min at room temperature after which absorbance was measured at 560 nm (*n* = 3). One unit (U) of SOD activity is defined as the quantity of enzyme that induces 50% inhibition of NBT photoreduction [26, 27].

### Statistical analysis

For technical replicates, all measures were based on the mean of three replica. One-way statistical analysis of variance (ANOVA) with confidence level at *p* ≤ 0.05 was used to statistically examine the collected data with the Post Hoc Duncan test at *p* ≤ 0.05 using COSTAT software version 6.3.

## Results and discussion

### Biosynthesis and characterization of SeNPs

**Fig. 1:**
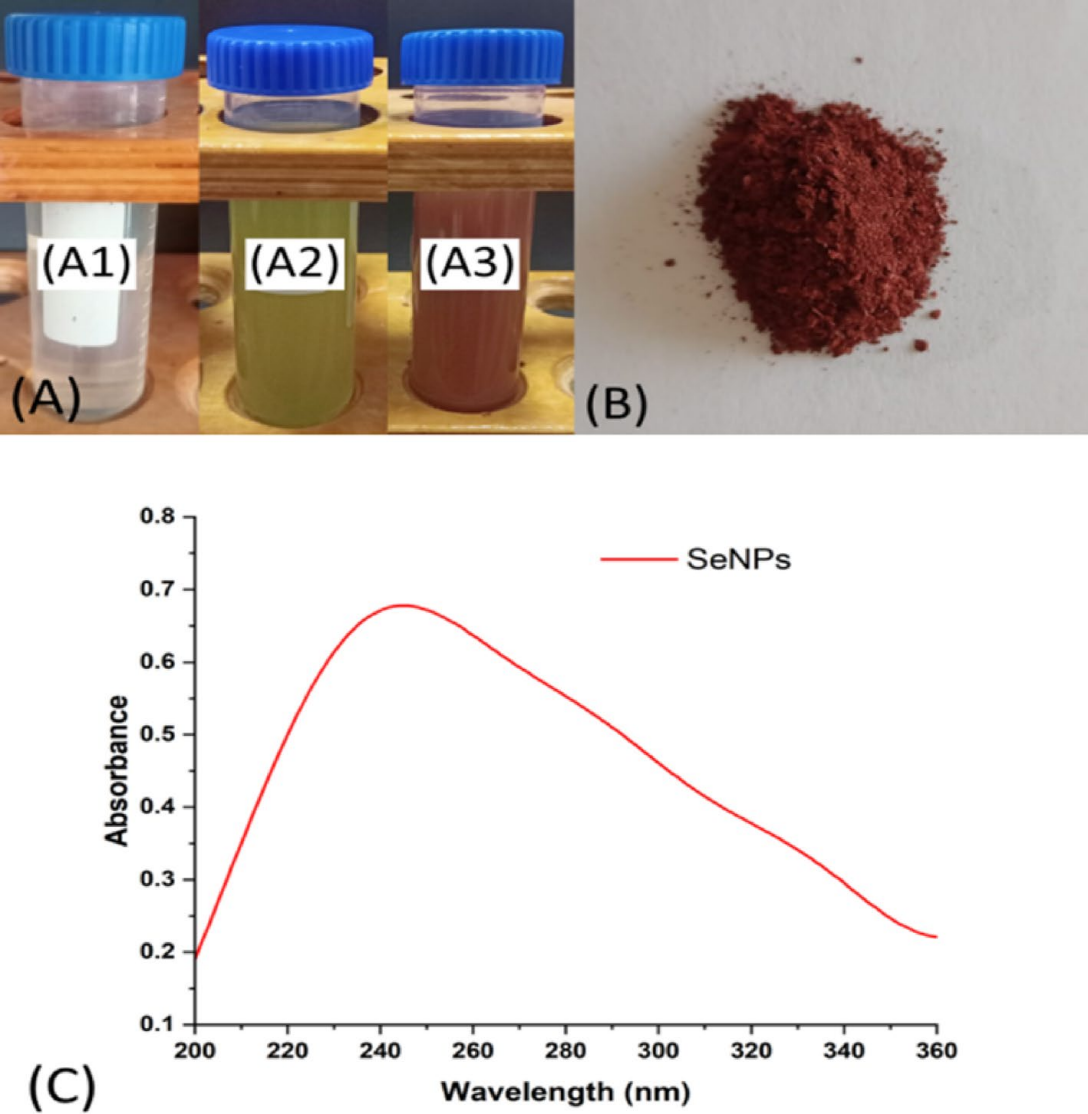
The color change represents the biosynthesis of SeNPs utilizing sodium selenite solution (A1), *Aloe vera* leaf extract (A2), and the biosynthesized SeNPs (A3), lyophilized selenium nanoparticles (**B**), and the UV-visible spectrum of the biosynthesized SeNPs (**C**)

This study focused on the eco-friendly production of SeNPs utilizing *Aloe vera* extract as the stabilizing and reducing agent. A solution of sodium selenite (Na_2_SeO_3_) was combined with an extract of *Aloe vera* leaf. In this work, the Na_2_SeO_3_ color was changed to brick red, indicating the biosynthesis of SeNPs after gradually adding *Aloe vera* extract (Fig. 1A). These results aligned with those of Vyas and Ran [20].

Biologically active constituents required for nanoparticles production are thought to be found in plant extracts at a specific concentration [28]. The capacity to produce SeNPs by plant extracts is significant since it minimizes downstream processing, and the challenges associated with managing cell cultures [29]. *Aloe vera*, a plant of great medicinal value, consists of peel (leaf) and gel. Several studies indicate that its leaf contains a variety of vitamins, proteins, lignin, polysaccharides, phenolic compounds, flavonoids, saponins, enzymes, sterols, and organic acids, which play an important role in the reduction of ions to their elemental form and the stabilization of synthesized nanoparticles [30]. The production of nanoparticles was indicated by the transformation of Na_2_SeO_3_ from colorless to brick-red during the reduction reaction [31] along with the spectrum of the generated nanoparticles at 247 maximum absorbance (Fig. 1C). This singular peak is representative of the typical spherical morphology of nanoparticles [32]. Previous research has found that green-synthesized SeNPs showed UV-visible maximum absorption in a variety of ranges. Utilizing fenugreek extract for the preparation of SeNPs revealed an absorbance peak between 200 and 400 nm, with the most significant peak at 390 nm [33]. Furthermore, SeNPs were produced from *Withania somnifera*, exhibiting a spherical shape and a maximum absorbance of 310 nm [34]. Production of SeNPs utilizing garlic cloves produced an absorption peak at 260 nm [35]. The green production of SeNPs was conducted utilizing *Melia azedarach* leaves, with a peak absorption detected at 263 nm by UV-visible spectroscopy [12]. SeNPs made with *Crataegus monogyna* extract showed the highest absorption peak at about 260 nm [36]. Likewise, Fesharaki et al. [37], the UV-visible maximum was observed at 218 and 248 nm (within the range of 200 to 300 nm) for SeNPs bio-synthesized by *Klebsiella pneumoniae*. Plant-based green synthesis is economically advantageous as it employs easily available and cost-effective plant resources [38, 39].

Another supporting evidence has come from surface plasmon resonance of the SeNPs, showing that the plant extract successfully produced SeNPs. Numerous reports have verified that the resonance peak of SeNPs is observed within the 200–800 nm range; however, the specific location is dependent on the local environment, particle size, shape, and material composition [40].

**Fig. 2:**
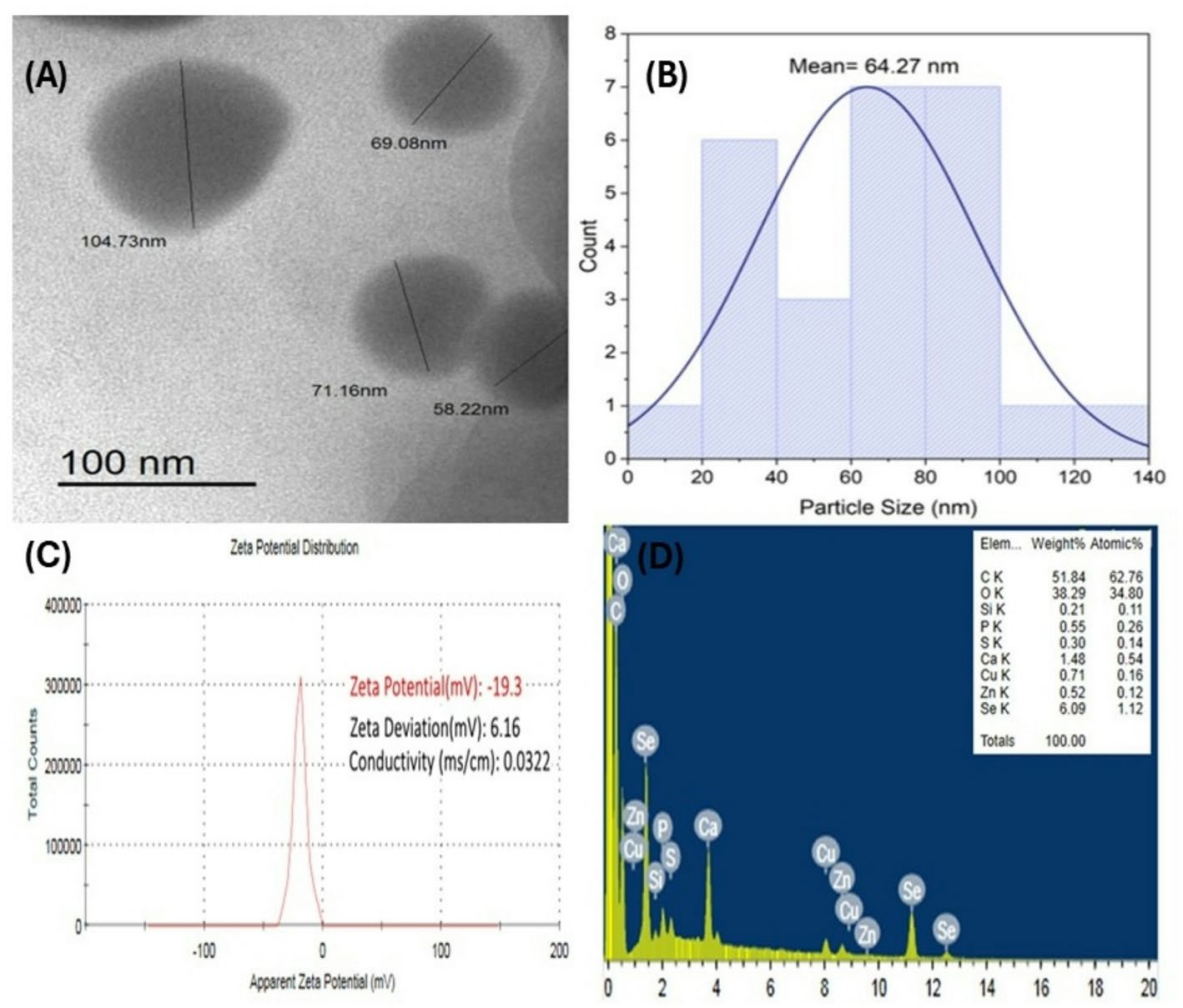
Biosynthesized SeNPs by *Aloe vera* leaf extract: transmission electron microscopy (**A**), particle distribution (**B**), zeta potential distribution (**C**), and energy-dispersive X-ray spectroscopy (EDX) analysis (**D**)

The morphological properties of *Aloe vera* leaf extract mediated SeNPs, including shape, aggregation, and size, are the primary factors influencing its biological efficiency and were studied by transmission electron microscopy (TEM) analysis. Figure 2A and B showed the spherical shape of synthesized SeNPs, which were regularly dispersed without agglomeration and exhibited sizes ranging from 21.48 to 104.73 nm. The size distribution histogram of SeNPs derived from TEM revealed an average particle size of 64.27 nm. The integration of nanoparticles in diverse applications depends mainly on characteristics like capping agents, surface charge, morphology, dimensions, and agglomeration [41].

SeNPs previously produced utilizing several plant extracts exhibited particle sizes ranging from *Withania somnifera* (45–90 nm) [34] and *Lycium barbarum* (125 nm) [42]. SeNPs synthesized by hawthorn fruit extract were estimated to be 113 nm in size [43]. The average size of SeNPs synthesized using stinging nettle extract was 107.7 nm [44]. Numerous reports have indicated that most SeNPs produced through green synthesis are spherical in shape [45]. These findings agree effectively with the current study. Also, the findings were similar to those of Matai et al. [46] and Gunti et al. [31], who documented the synthesis of spherical SeNPs from plant extracts.

The stability of the obtained SeNPs was examined using zeta potential, which determines the electric charge on the surface of the nanoparticles. SeNPs have a zeta potential of − 19.3 mV, shown by a singular peak in Fig. 2C. High positive or negative zeta potentials in suspensions induce repulsion among particles, therefore inhibiting aggregation. With a polydispersity index (PDI) of 0.206, the nanoparticles showed a uniform size distribution. The negative charge potential resulted from the reducing agent oxidizing the polyphenolic and flavonoid constituents of the leaf extract. Previously, when zeta potential of all particles in a suspension was either negative or positive, they exhibited a tendency to repel one another, leading to a significant decrease in their tendency to aggregate [47]. The observed strongly negative zeta potential values indicates significant physical stability in water caused by the electrostatic interactions among the synthesized nanoparticles, as verified by previous studies [48]. The zeta potential of Phyto-fabricated SeNPs produced a peak of − 24.4 mV, proving that PF SeNPs surface is negatively charged [31]. Biosynthesized SeNPs by probiotic *Bacillus subtilis* BSN313 exhibited a zeta potential of − 26.9 mV [49]. An Energy-dispersive X-ray (EDX) analysis was utilized to examine the elemental composition of SeNPs. It revealed the presence of carbon (51.84), oxygen (38.29 wt%), selenium (6.09 wt%), and trace elements including Si, P, S, Ca, Cu and Zn (0.21, 0.55, 0.3, 1.48, 0.71, and 0.52% respectively) Fig. 2D. In this study, the additional elements observed in the EDX chromatogram with SeNPs may be incorporated from the constituents of *Aloe vera* extract. This result aligns with the findings of Wadhwani et al. [50] and El-Saadony et al. [51].

The EDX analysis of SeNPs mediated by *V. arctostaphylos* L. fruit extract demonstrated a powerful selenium signal at approximately 2.5 keV. X-ray emissions due to carbohydrates, proteins, and enzymes present in the extract [42, 48]. Reports indicate that, during biosynthesis by plants, phytochemicals encapsulate the surface of SeNPs, SeNPs, which are derived from green, orange peel extract, are particularly composed of polyphenolic components acting as reducing agents [52]. The concentration of each element in the sample is directly correlated to the intensity of X-ray signals in the EDXS spectrum. It corresponds with the results of Barzegarparay et al. [36] and Puri and Patil [47]. Phytochemicals were mostly identified in SeNPs produced by plant extracts [12, 34, 53]. These phytochemicals may be crucial in the biological functions of the SeNPs [53].

**Fig. 3:**
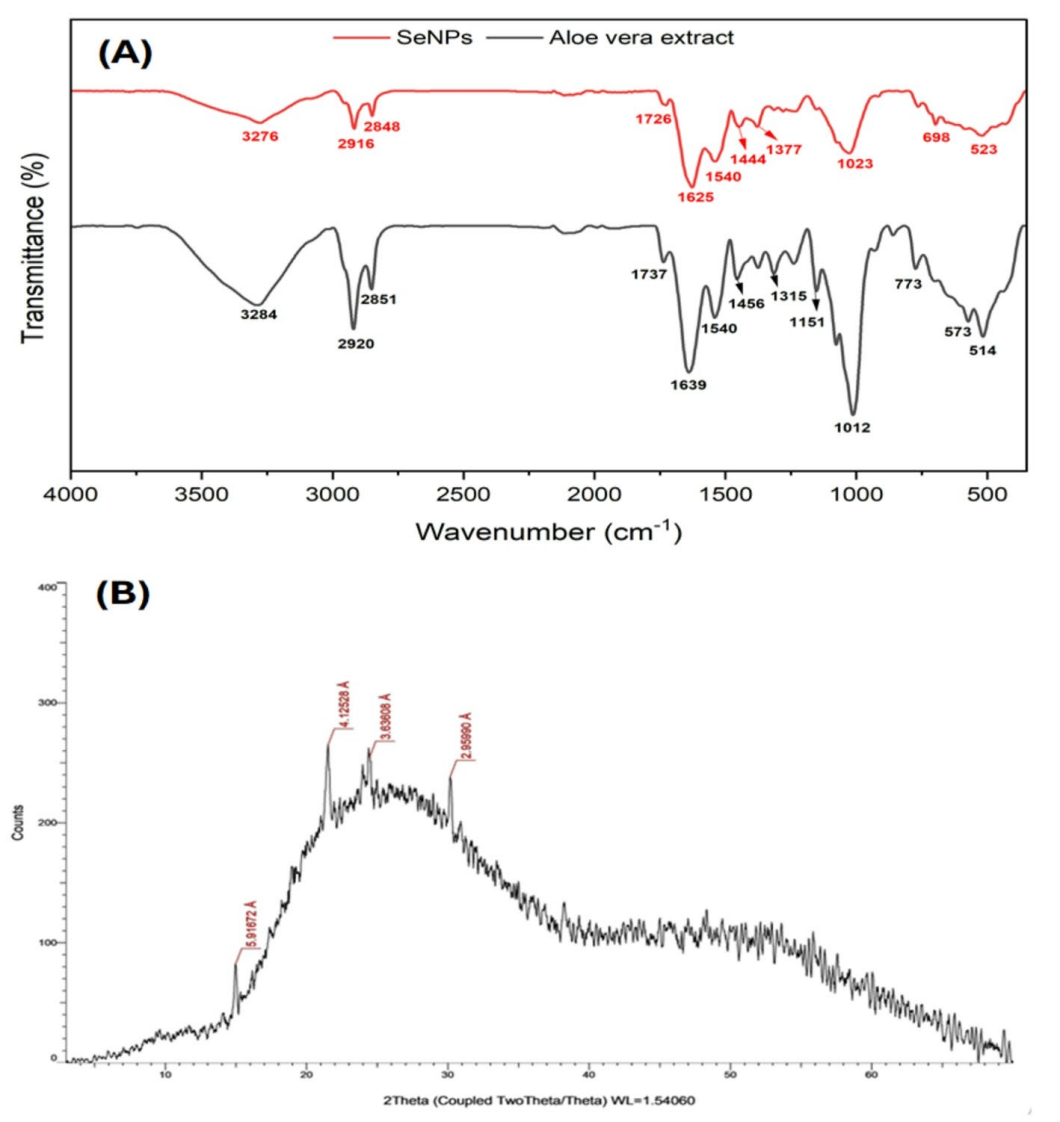
FTIR spectroscopy of *Aloe vera* leaf extract and the resulting SeNPs (**A**), and X-ray diffraction patterns of bio-synthesized SeNPs (**B**)

The FTIR spectrum (Fig. 3A) of *Aloe vera* leaf has presented multiple peaks at 3284, 2920, 2851, 1737, 1639, 1540, 1456, 1315, 1151, 1012, 773, 573 and 514 cm^− 1^, corresponding to attendance of–OH group, stretching vibration of aliphatic C–H, carboxylic acid O–H, carbonyl C = O stretching, amide I vibrations, amide II band, CH_2_ bending, phenolic C–O stretching, C–O–C stretching, polysaccharide C–O, C–H out-of-plane bending, C–C–O bending in carbohydrate structures and C–O skeletal bending, respectively. This analysis indicated the function groups behind the synthesis process of SeNPs. Whereas SeNPs showed peaks at 3276, 2916, 2848, 1726, 1625, 1540, 1444, 1377, 1023, 698 and 523 cm^− 1^ peaks when subjected to the same analysis. The data obtained corroborated the findings of Vyas and Rana [20].

The observed peaks of SeNPs were like those of the extract, with a little shift [54]. The presence of alcohol and phenol in U. dioica extract was indicated by the spectral lines at 3259 cm^− 1^, which correspond to O–H stretching vibrations. Studies have demonstrated that phytochemicals act as stabilizers during the SeNPs synthesis [44]. According to the present study, the band at 1639 in *Aloe vera* extract shifted to 1625 in SeNPs due to the presence of proteins that bind to SeNPs via amine groups. The band at 1620.34 cm^− 1^ in the fruit extract was associated with amide I vibrations, whereas the band in SeNPs shifted to 1600.16 cm^− 1^ because of proteins that may be bound to SeNPs via amine groups [48]. The presence of these groups may indicate that they serve a reducing function in the synthesis of SeNPs, as Se interacts with the -OH group through hydrogen bonds [55].

Results demonstrate that biomolecules from the leaf extract significantly contributed to the production of the SeNPs by coating their surface [53]. The presence of extra peaks in the SeNPs’ FTIR spectra could be the result of interactions between sodium selenite and metabolites in the extract through the reduction and capping of the produced SeNPs. The peaks between 400 and 800 cm^− 1^ correspond to the bending and stretching of Se-O, resulting from the interaction of SeNPs with carbonyl groups, this eventually creates a coating layer that prevents agglomeration and aggregation around the surface of SeNPs [56].

The XRD examination confirmed the existence of SeNPs in the sample. The results indicated that the produced SeNPs exhibited lower crystallinity and were largely amorphous **(**Fig. 3B). The spectra displayed slight reflections at angles of 14.96, 21.52, 24.46, and 30.17, indicating elemental selenium’s presence. The result obtained aligned with the findings of Sharma et al. [57], which showed two distinct peaks at an angle of 2θ, specifically at 24.2° and 30.3°. The results of XRD showed that SeNPs made by *Pseudomonas stutzeri* had an amorphous structure [58]. Also, Jiang et al. [59] observed the amorphous feature of SeNPs. Plant extracts have been shown to produce amorphous SeNPs in many earlier studies [53, 60].

The mean size of the SeNPs was calculated using Scherrer’s equation D = (0.89λ/βCOSθ), where D represents the mean size of the SeNPs, λ is the wavelength (0.15418 nm), β indicates the full width at half maximum (FWHM) peak, and θ specifies the Bragg diffraction angle [61]. The mean size of SeNPs as determined by XRD analysis was 68.94 nm. This size agreed well with the TEM results.

When taken as a whole, these analyses show effective and controlled SeNP synthesis: UV-Vis confirmed SeNP formation at 247 nm, TEM showed uniform spherical shape SeNPs with mean size 64.27 nm, FTIR showed *Aloe vera* phytochemicals peaks that mediated reduction and stabilization and shifted as a result of SeNPs synthesis, EDX confirmed elemental selenium, and XRD confirmed lower crystallinity and high amorphous structure.”

### Biological activities of the obtained SeNPs

#### Antibacterial activity

**Fig. 4:**
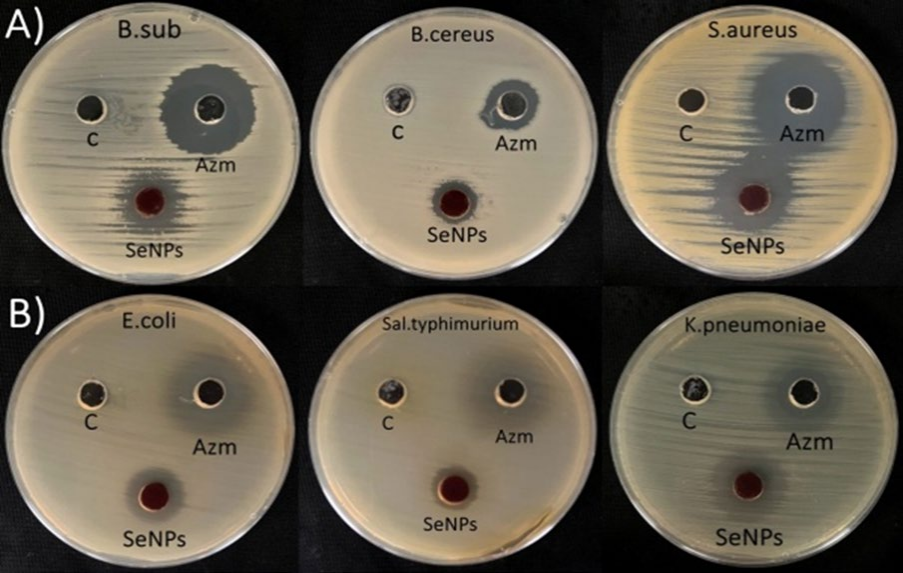
Antibacterial activity of *Aloe vera* extract mediated synthesized SeNPs, Azithromycin (+ ve control), and DMSO (-ve control) by well diffusion method against** A** Gram-positive bacteria (*Bacillus subtilis*,* Bacillus cereus*,* Staphylococcus aureus)*,** B** Gram-negative bacteria (*Escherichia coli*,* Salmonella typhimurium*,* Klebsiella pneumonia)*

The antibacterial efficiency of SeNPs synthesized by *Aloe vera* extract against various pathogenic bacterial strains was examined. The growth of all examined bacteria either gram-negative or gram-positive was inhibited by SeNPs at a concentration of 15 mg mL^–1^ (Fig. 4).

In the current study, the effect of the biosynthesized SeNPs to inhibit the growth of pathogenic bacteria; *Escherichia coli*, *Klebsiella pneumonia*, *Salmonella typhimurium*, *Bacillus cereus*, *Bacillus subtilis*, and *Staphylococcus aureus* were investigated by well diffusion method and showed 17 ± 0.46, 24 ± 0.58, 14 ± 0.69, 14 ± 0.35, 20 ± 0.29, 25 ± 0.23 mm respectively (Table 1). The most significant inhibition was seen in *Staphylococcus aureus* (25 mm), followed by *Klebsiella pneumoniae* (24 mm), *Bacillus subtilis* (20 mm), *Escherichia coli* (17 mm), and *Salmonella typhimurium* and *Bacillus cereus* (both 14 mm). These results clearly demonstrated strain-dependent variations in antimicrobial sensitivity to SeNPs. Azithromycin was used as the positive control, while DMSO served as the negative control. *Staphylococcus aureus*,* Klebsiella pneumonia*, and *Bacillus subtilis* were the most affected strains as indicated by their highest inhibitory zones.

**Table 1:** Antibacterial activity of *Aloe vera* leaf extract mediated SeNPs

Organisms name	Diameter of inhibition zone (in mm)		
Gram-positive bacteria	SeNPs	Azithromycin (positive control)	DMSO (negative control)
*Bacillus subtilis*	20.00^**c**^±0.29	27.00^**a**^±0.58	0.00 ± 00
*Bacillus cereus*	14.00^**e**^±0.35	16.00^**c**^±0.87	0.00 ± 00
*Staphylococcus aureus*	25.00^**a**^±0.23	27.00^**a**^±0.40	0.00 ± 00
Gram-negative bacteria			
*Escherichia coli*	17.00^**d**^±0.46	18.70^**b**^±0.12	0.00 ± 00
*Salmonella typhimurium*	14.00^**e**^±0.69	14.50^**d**^±0.29	0.00 ± 00
*Klebsiella pneumonia*	24.00^**b**^±0.58	16.00^**c**^±0.92	0.00 ± 00

Data are presented as mean ± standard error (mean ± SE; *n* = 3). Values within the same column labeled with different superscript letters (a–e) are significantly different (p ≤ 0.05)

 Fardsadegh and Jafarizadeh-Malmiri [62] revealed that SeNPs generated by *Aloe vera* leaf extract showed antibacterial efficacy against *S. aureus* and *E. coli*, the inhibitory zones surrounding *S. aureus* had a larger diameter (12 mm) than those surrounding *E. coli* (10 mm). Meanwhile, the synthesized SeNPs using the *Withania somnifera* aqueous extract exhibited antibacterial efficacy against *S. aureus*,* B. subtilis*, and *Klebsiella pneumoniae* with no observable activity against *E. coli* [38].

Biogenic SeNPs treatments might lead to oxidative damage that would damage the bacterial cell membranes’ permeability leading to the efflux of polysaccharides and proteins and finally resulting in cell death [63]. Additionally, SeNPs have been shown to alter cellular respiration and decrease the transmembrane capacity by destroying the integrity of the cell membrane and inhibiting the activity of essential enzymes for bacterial growth and metabolism particularly dehydrogenases [64]. SeNPs may exhibit antibacterial activity because of the inhibition of enzymes or the production of reactive oxygen species, potentially leading to microbial cell death [53, 65].

#### Antioxidant activity

**Fig. 5:**
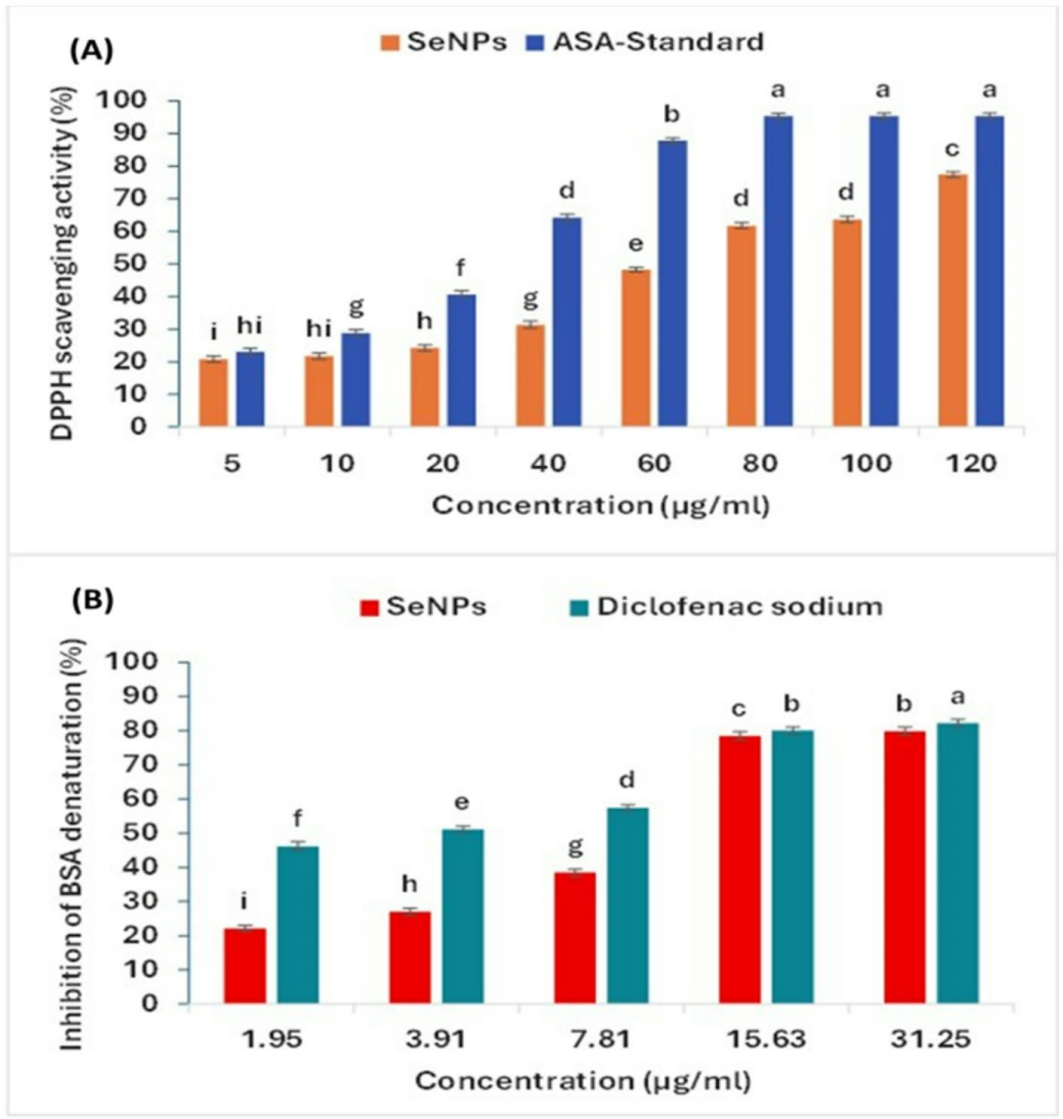
Antioxidant activity of SeNPs using DPPH scavenging activity compared with standard ascorbic acid (**A**), and anti-inflammatory activity of SeNPs compared with Diclofenac sodium as a standard (**B**), Data with different letters are significantly different (*p* ≤ 0.05). Bars represent standard error (SE)

The antioxidant ability of SeNPs was tested by DPPH free radical scavenging assay (Fig. 5A). The results showed that by increasing the concentration from 5 to 120 µg mL^–1^, the antioxidant activity increases from 20.75 ± 0.95 to 77.39 ± 0.90% and from 23.00 ± 1.05 to 95.34 ± 0.95% for SeNPs and ascorbic acid respectively. The inhibitory concentration for 50% of DPPH radical (IC_50_) was determined as 66.86 and 30.51 µg mL^–1^ SeNPs and ascorbic acid, respectively, which are showed in the Supplementary Material (Fig. S1).

The activity is exactly proportional to the concentration of green-produced SeNPs [66]. The antioxidant activity results indicated higher scavenging ability for biogenic SeNPs (IC_50_ 83.89 ± 2.11 µg mL^–1^) compared to SeNPs which were produced chemically (IC_50_ 174.79 ± 0.29 µg mL^–1^) [67].

SeNPs mediated by *Moringa oleifera* demonstrated concentration-dependent DPPH scavenging activity between 15.63 and 1000 µg/mL, reaching 84% at the highest dosage (1000 g/mL), which is equivalent to ascorbic acid’s 91% at the same dose [68], and another investigation that used *Tinospora cordifolia* SeNPs, which demonstrated a 78% scavenging activity at 100 µg/mL [69]. Multiple studies suggested that efficient biological application for SeNPs as effective antioxidants, particularly due to their redox modulatory characteristics [47, 70].

The sources of the scavengers of free radical are either natural, including tannins, phytoestrogens, flavonoids, and phenolics or synthetic, for example nanomaterials [71]. Metal-oxide and metal nanoparticles are distinguished by their capacity to serve as scavengers for free radical [72]. In vivo conditions, the antioxidant activity of SeNPs may be attributed to their capacity to improve selenoenzymes activity, including glutathione peroxidase [73]. The antioxidants of NPs may result from neutralization and prevention of DPPH free radical production by transfer of electron [66, 74]. Furthermore, the distinctive characteristics of nanoparticles, particularly their enhanced surface-to-volume ratio, may improve antioxidant activity [66, 75].

Ultra-small diameter lentinan Se nanoparticles SeNPs demonstrated better antioxidant qualities because of their reduced particle size and nanoscale dimensions [76]. The functionalized nanoparticles (IONP@AO) were found to be four times more effective in scavenging free radicals than unfunctional IONP in the DPPH test [77].

#### Inhibition of protein denaturation by SeNPs

The substance’s capacity to prevent protein denaturation indicates its potential for anti-inflammatory activity. The obtained SeNPs were examined for anti-inflammatory properties utilizing bovine serum albumin (BSA). SeNPs exhibited an enhanced inhibitory percentage as the concentration increased from 1.95 to 31.25 µg mL^–1^, the BSA denaturation inhibition activity of the synthesized SeNPs showed 79.87 ± 1.21%, whereas the standard exhibited 82.23 ± 1.13% inhibition. The reagent for this experiment was BSA (Fig. 5B). The protein denaturation IC_50_ values were found to be 12.50 µg ml^− 1^ for SeNPs and 1.59 µg ml^− 1^ for diclofenac, which (Fig. S2). Bovine serum albumin constitutes almost 60% of the total protein content in animal serum [78].

SeNPs demonstrated higher bioavailability due to their increased catalytic efficiency, high adsorption ability, and less toxicity. Furthermore, it has demonstrated the anti-inflammatory and antioxidant capacity of SeNPs produced from *T. vulgaris* [79, 80]. Selenium acts as an effective anti-inflammatory agent [81]. Consequently, the findings of our study align with similar research, demonstrating a significant ability to scavenge free radicals that induce damage and inhibit protein denaturation. It may serve as a novel and environmentally sustainable anti-inflammatory agent for future disease treatment [82]. Numerous studies have demonstrated that selenium can decrease the inflammatory response in the body resulting from autoimmune disorders or inflammatory diseases [81]. The mRNA expression of pro-inflammatory cytokines, such as inducible NO synthase (iNOS), TNF-α, and interleukin IL-1, was observed to be downregulated by SeNPs, thereby decreasing inflammation [81, 83].

The NFkB pathway is responsible for the induction of inflammation by enhancing the expression of numerous proinflammatory cytokines, such as TNF alpha and IL-6. Pro-inflammatory cytokines expression was significantly reduced when selenium was provided as a supplement. Because selenium incorporates selenoproteins, it significantly improves their antioxidant function, resulting in a reduction in inflammatory responses in autoimmune and inflammatory diseases [81, 84].

The most significant blood proteins, including human serum albumin (HSA), human hemoglobin (HHb), and cytochrome c (Cyt c), can bind to nano-selenium. The findings demonstrated that these proteins’ secondary structure is unchanged [56]. Additionally, *Aloe vera* polysaccharides upregulate anti-inflammatory cytokines like IL-10 while downregulating proinflammatory cytokines including IL-1β, IL-6, and TNF-α [85]. Also, polysaccharides prevent neutrophils and monocytes from migrating to inflammatory tissues, minimizing tissue damage and encouraging the resolution of inflammation [86, 87].

#### Anticancer and biocompatibility

**Fig. 6:**
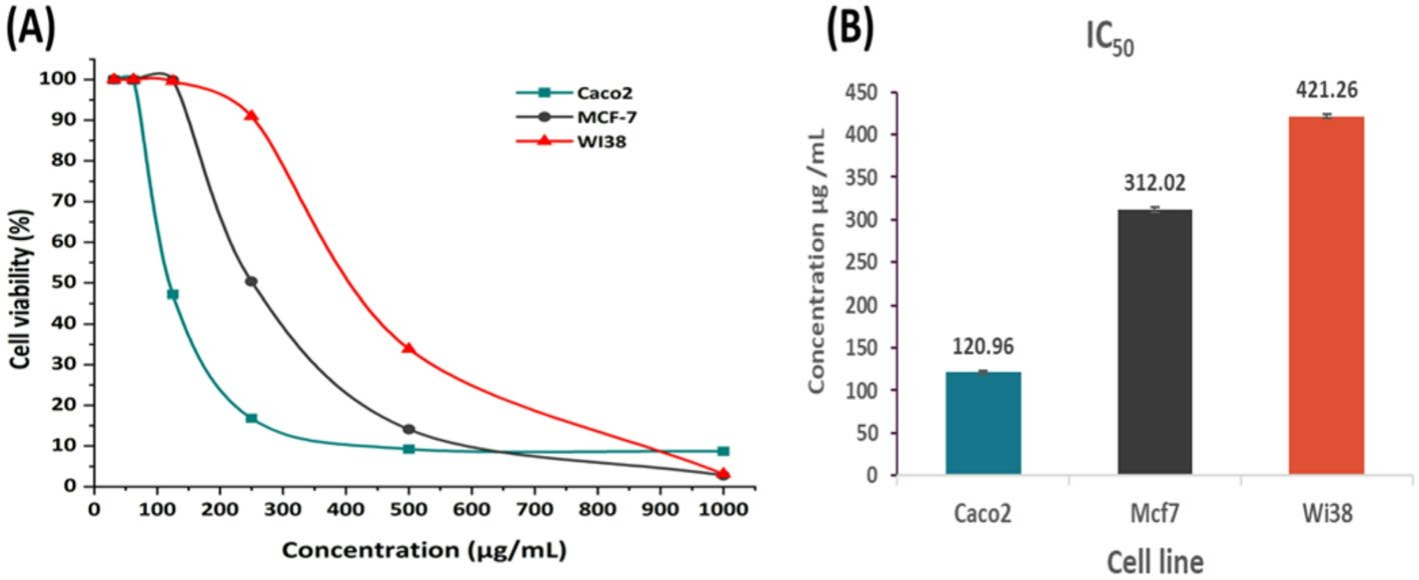
Cytotoxic activity of SeNPs on two cancer cell lines (Caco2, MCF7) and normal cell line (WI38) (**A**) and calculated IC_50_ values (**B**), Error bars represent standard error

Green synthesized SeNPs were examined for their anticancer activity against MCF7 and Caco2 cancer cells, and their biocompatibility against a normal cell, WI38. The MTT assay method was used to estimate the impacts of SeNPs treatment on cellular proliferation and cell viability (Fig. 6A). Concentration-dependently, SeNPs showed significant anticancer effects against cancer cell lines that have been tested. The IC_50_ of SeNPs was determined to be 120.96 ± 1.87, 312.02 ± 3.25, and 421.26 ± 2.26 µg mL^–1^ for caco2 (showed better anti-tumor effect), MCF7(Showed least anti-tumor effect), and WI38, respectively. IC50 values (Fig. 6B), and various cell line photos are presented in the Supplementary Material (Fig. S3).

The viability of normal and cancer cell lines was not significantly affected by SeNPs at small concentrations (≤ 62.5 µg mL^–1^). The cell viability of all cells was within the range of 99.86–100%. However, the viability of Caco2 was 47.23% at a concentration of 125 µg mL^–1^, which is lower than that of the cancer cell line MCF7 (99.86%) and the normal cell line WI38 (99.56%). By increasing the concentration of SeNPs to 250 µg mL^− 1^, the viability of the Caco-2 cancerous cell line was significantly reduced, reaching 16.83% and 50.36% for MCF7, respectively, in comparison to the viability of the WI38 normal cell line, which was 90.94%.

Multiple studies have investigated SeNPs as an anticancer agent used against various cancerous cell lines [44, 88]. In accordance with the results found, the effect of phyto-synthesized SeNPs depended upon the concentrations toward cancer cell lines IMR-32, Caco-2, and MCF-7 and the normal cell line Vero [71]. HepG2 cells were subjected to dose-dependent cytotoxicity by SeNPs, which caused apoptosis as seen by a significant increase in the percentage of apoptotic cells [89]. During a sub-chronic period, gold nanoparticles exhibit dose-dependent cytotoxicity, mainly due to their uptake and accumulation in mitochondria, which causes oxidative stress and death [90].

This was in accordance with previous studies that had shown that SeNPs exhibit a significant level of selective toxicity toward cancer cells [8, 89], and only minimal side effects on normal cells [91]. The material/compound is classified as non-toxic if the IC_50_ is greater than or equal to 90 µg mL^− 1^ [92]. The IC_50_ of *Penicillium verhagenii* mediated SeNPs was examined to be 283.8 ± 7.5, 225.7 ± 3.6, 472.8 ± 5.8, and 454.8 ± 29.9 µg mL^–1^ for MCF7, PC3, Vero, and WI38 cell lines, respectively [66]. In comparison to normal cell lines, the data obtained indicated that SeNPs had a target orientation toward cancerous cell lines at small concentrations [66]. The SeNPs produced by Gossypium barbadense showed the highest cytotoxic activity, and HCT116 was more sensitive than PANC1. HCT116 cells showed IC50 values of 99.41 ± 1.87 and 240.79 ± 6.56 µg/mL, while PANC1 cells had IC50 values of 358.54 ± 3.83 and 349.43 ± 7.76 [93].

The studies suggested that SeNPs have a variety of anti-cancer mechanisms, including the production of ROS, DNA fragmentation, mitochondrial dysfunction, cellular homeostasis disruption, and cell apoptosis [71]. The most focus is given to apoptosis, which is typified by nuclear chromatin condensation, cytoplasmic compression, and membrane malfunction. The primary cytotoxicity mechanisms caused by SeNPs were discovered to be oxidative stress and ROS production, where ROS influences apoptosis by controlling the enzyme activity involved in cell death pathways [94].

According to Wang et al. [95], The activities of SeNPs as anticancer agent may result from their capacity to stimulate apoptosis in cancer cells, resulting in cellular degeneration and eventually the death of cells. The cytotoxicity of nanoparticles is mainly because of their extensive surface area, facilitating effective drug delivery [47].

#### Effect of SeNPs on the antioxidant markers (CAT and SOD enzymes) in Caco2 and MCF-7 cell lines

**Fig. 7:**
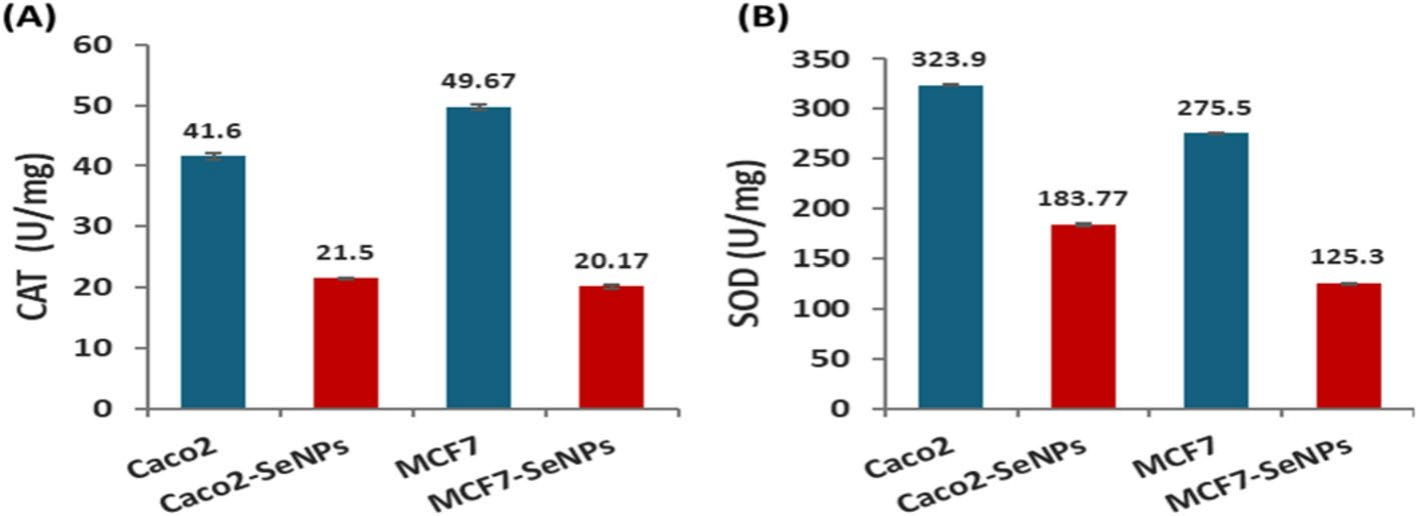
Cancer cell lines Caco2 and MCF7 were treated with SeNPs then antioxidants markers CAT (**A**), and SOD (**B**) were determined, Error bars represent standard error

As shown in Fig. 7A&B, CAT enzyme activity significantly decreased from 41.6 ± 0.52 to 21.5 ± 0.28 U/mg and from 49.67 ± 0.44 to 20.17 ± 0.26 U/mg in Caco2 and MCF-7 cell lines, respectively. Also, SOD enzyme activity decreased from 323.9 ± 0.85 to 183.77 ± 0.92 U/mg and from 275.5 ± 0.75 to 125.3 ± 0.52 U/mg in Caco2 and MCF-7 cell lines, respectively. Percent of change was calculated, which showed a decrease in CAT activity of 48.32% and 59.39%, for SOD also showed a decrease of 43.26% and 54.52% for Caco2 and MCF-7 cell lines, respectively. These results agreed with the cytotoxicity results.

According to the current findings, an additional investigation indicated the activity of SOD and GPx enzymes were significantly reduced after treatment with SeNPs in IPEC-J2 cells [96, 97]. Biosynthesized SeNPs produced by yeast exhibited a reduction in CAT and SOD enzyme activity, decreasing from 0.79 ± 0.04 to 0.24 ± 0.06 U/mg and from 7.8 ± 0.3 to 2.89 ± 0.13 U/mg, respectively [8]. The activity of the CAT and SOD enzymes was markedly decreased in groups exposed to SeNPs compared to the untreated cells [83].

Although ROS play a variety of signaling and informational roles, too much of them can weaken the antioxidant defense system, damaging proteins, lipids, and DNA [98]. The two antioxidant enzymes SOD and CAT are important for maintaining the amount of ROS in organisms and are employed as bioindicators of elevated ROS generation [99]. Superoxide dismutases (SODs), which break down superoxide radicals to prevent oxidative stress, are the first line of defense against damage caused by reactive oxygen species (ROS) [100].

The unique characteristics of biosynthesized SeNPs, such as high surface area, biocompatibility, and ability to target specific cells, make them an attractive option for medical field. Future studies could explore the application of the developed nanoparticles in plant systems, such as targeted drug delivery, growth regulation, or stress tolerance enhancement. Comparative studies on different plant species or tissues could provide deeper insight into mechanisms of uptake and bioactivity, paving the way for agricultural or environmental applications.

## Conclusion

Our findings indicate that fresh leaf extract from *Aloe vera* is recommended since it includes numerous essential bioactive compounds that are used as reducing agents and stabilizing agents in the environmentally friendly biogenic synthesis of SeNPs. EDX, FTIR, TEM, zeta potential, UV-vis spectroscopy, and XRD studies are used to characterize the biosynthesized SeNPs.

SeNPs have significant biomedical uses as antibacterial agents against different pathogenic bacterial strains both Gram-negative and Gram-positive and *S.* *aureus* (25 mm), *K.* *pneumoniae* (24 mm), and *B.* *subtilis* (20 mm) had the most effects, they also exhibit antioxidant action by scavenging DPPH; they have anti-inflammatory activity by inhibiting the denaturation of BSA proteins; they have anticancer properties against MCF7 and Caco2 cancer cell lines by reducing their antioxidant enzymes, CAT and SOD, with a stronger inhibitory effect on Caco2 cells, suggesting a cytotoxic response that was dependent on the cell line, and they have no cytotoxic effect on the normal cell line, WI38.

## Supplementary Information

Below is the link to the electronic supplementary material.

Supplementary Material 1.

## Data Availability

All data used in the current study are available within the manuscript.

## Abbreviations

BSA: Bovine serum albumin
CAT: Catalase
DMSO: Dimethyl sulfoxide
DPPH: 2,2-diphenyl-1-picrylhydrazyl
EDX: Energy-dispersive X-ray spectroscopy
FTIR: Fourier transform infrared
GPx: Glutathione peroxidase
MTT: 3-(4,5-dimethylthiazol-2-yl)-2,5-diphenyltetrazolium bromide
NBT: Nitroblue tetrazolium
ROS: Reactive oxygen species
SeNPs: Selenium nanoparticles
SOD: Superoxide dismutase
TEM: Transmission electron microscopy
XRD: X-ray diffraction
