# Correction: Biosynthesis of selenium nanoparticles by *Aloe vera* leaf extract and its biomedical applications

**DOI:** 10.1186/s11671-026-04572-z

**Published:** 2026-05-08

**Authors:** Aya M. El-Ebidy, Amr M. Mowafy, Heba M. M. Abdel-Aziz

**Affiliations:** 1 Botany Department, Faculty of Science, Mansoura University, Mansoura, Egypt; 2 Department of Biological Sciences, Faculty of Science, New Mansoura University, New Mansoura City, Egypt

**Correction to: Discover Nano (2026) 21:91**

10.1186/s11671-026-04489-7

Following the publication of the original article [1], it was noted that the author’s name Heba M. M. Abdel-Aziz was incorrectly written as Heba M. Abdel-Aziz.

The original article [1] has been corrected.
