# Supplementary material for: Biosynthesis of selenium nanoparticles by Aloe vera leaf extract and its biomedical applications
Source: Discov Nano. 2026 Mar 30;21(1):91. doi: 10.1186/s11671-026-04489-7 (PMC13036000; doi:10.1186/s11671-026-04489-7)
Supplement: Supplementary file 1 — Supplementary Material 1. [file 11671_2026_4489_MOESM1_ESM.docx]

Supplementary Material


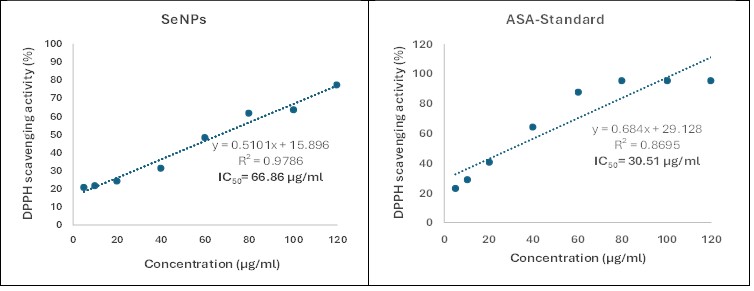


**Figure S1: DPPH radical scavenging IC_50_ values of Selenium nanoparticles (SeNPs) and ascorbic acid.**


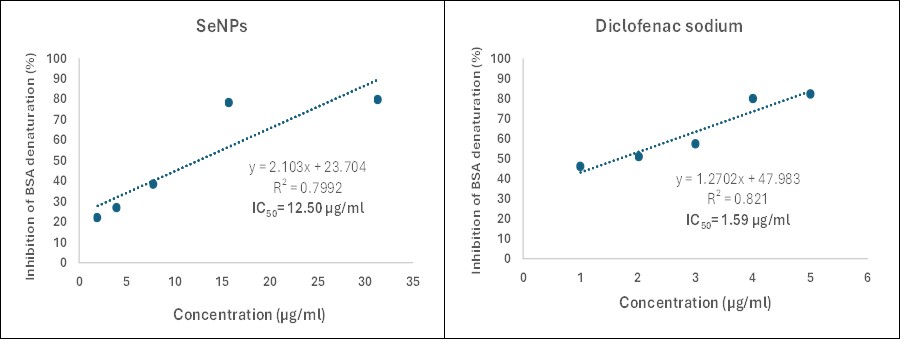


**Figure S2: Bovine serum albumin (BSA) denaturation inhibition IC_50_ values of Selenium nanoparticles (SeNPs) and diclofenac sodium.**

| 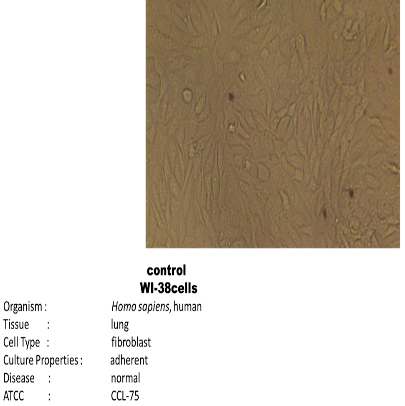WI38- control | 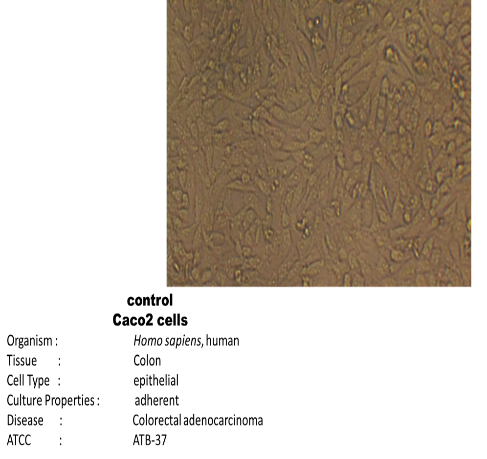Caco2- control | 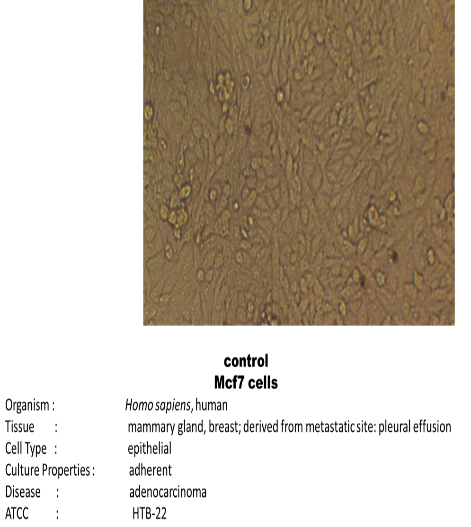MCF7- control |
| --- | --- | --- |
| 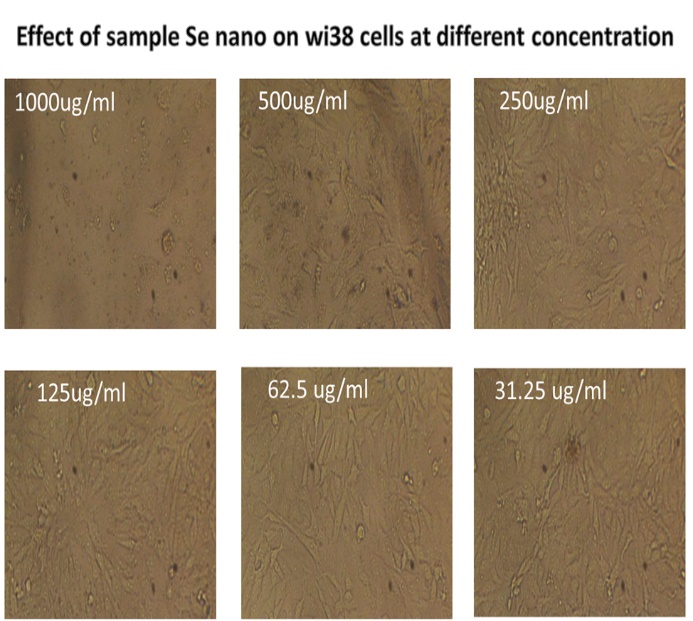 | 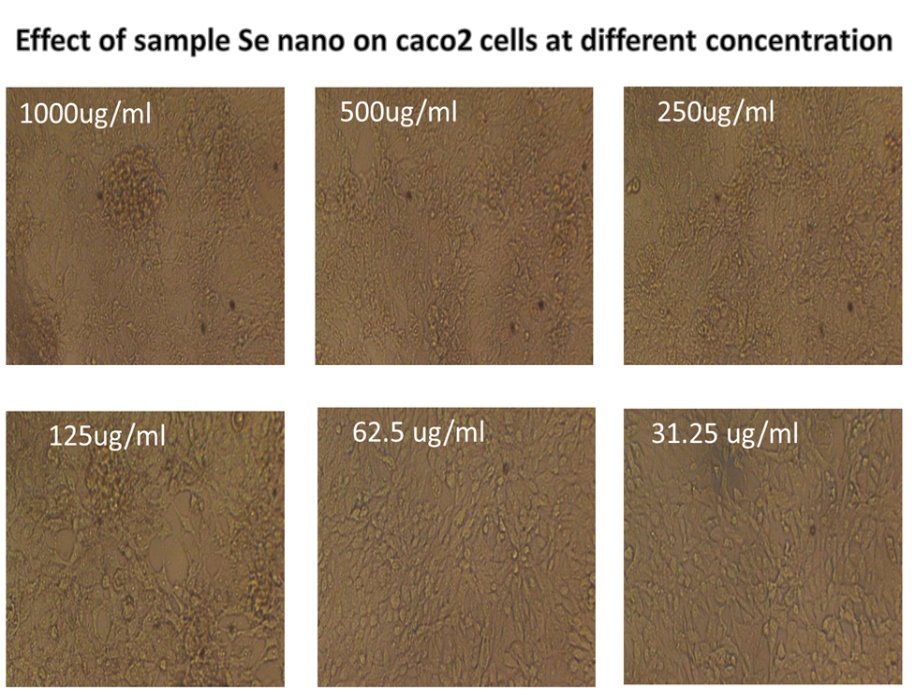 | 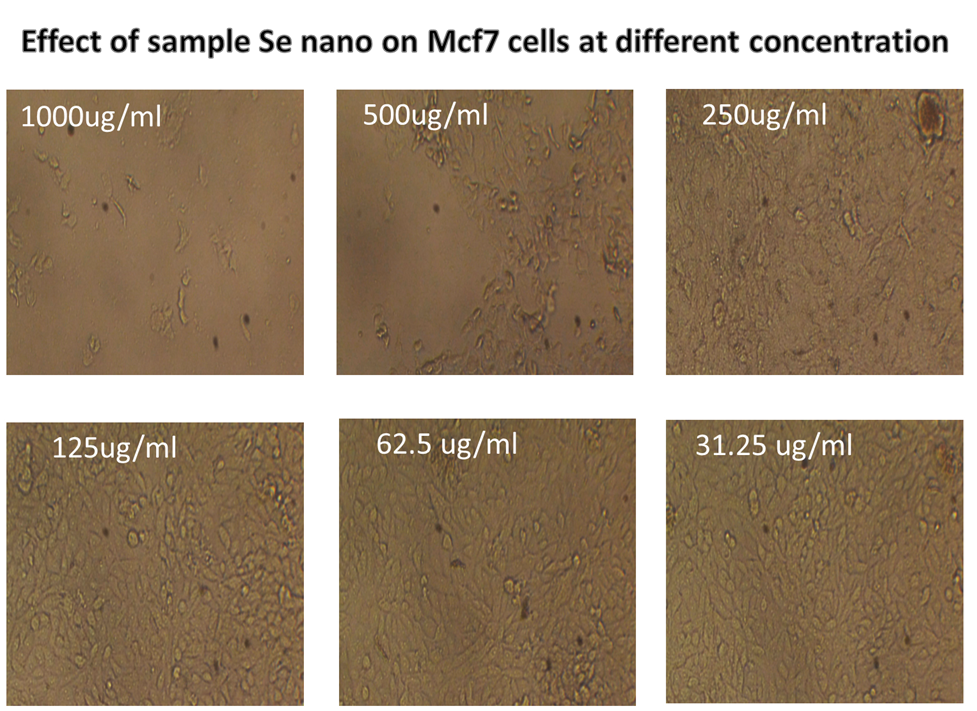 |
| 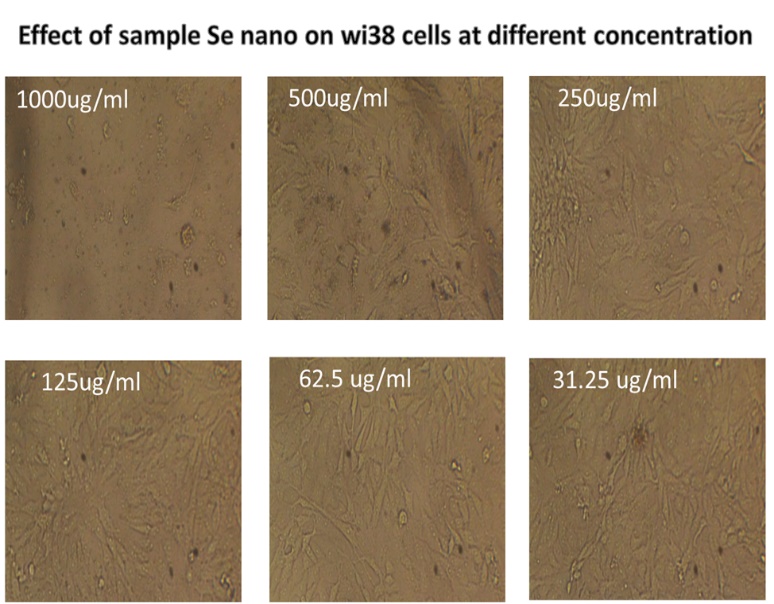 | 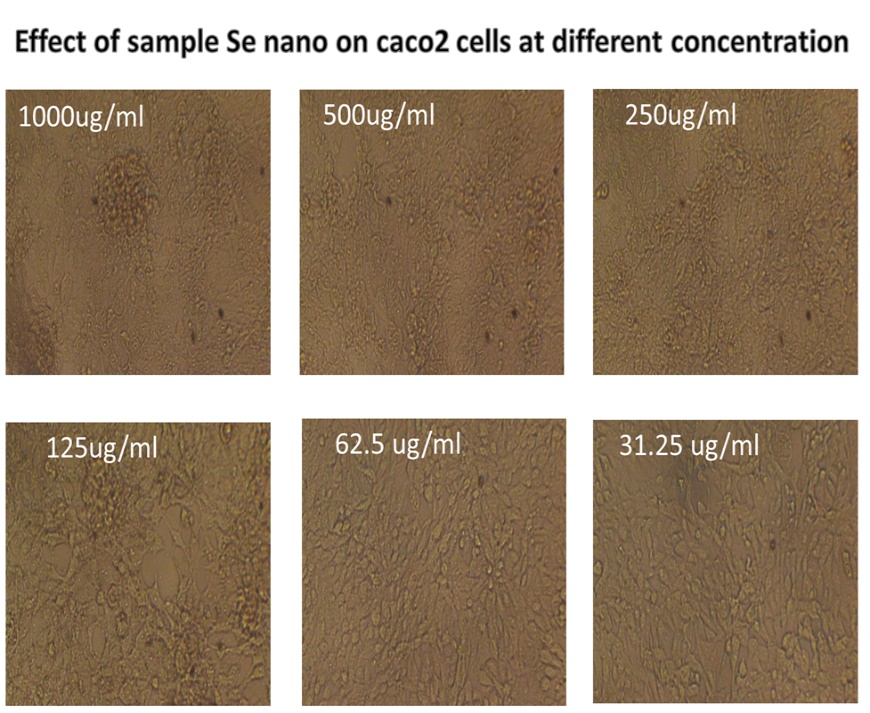 | 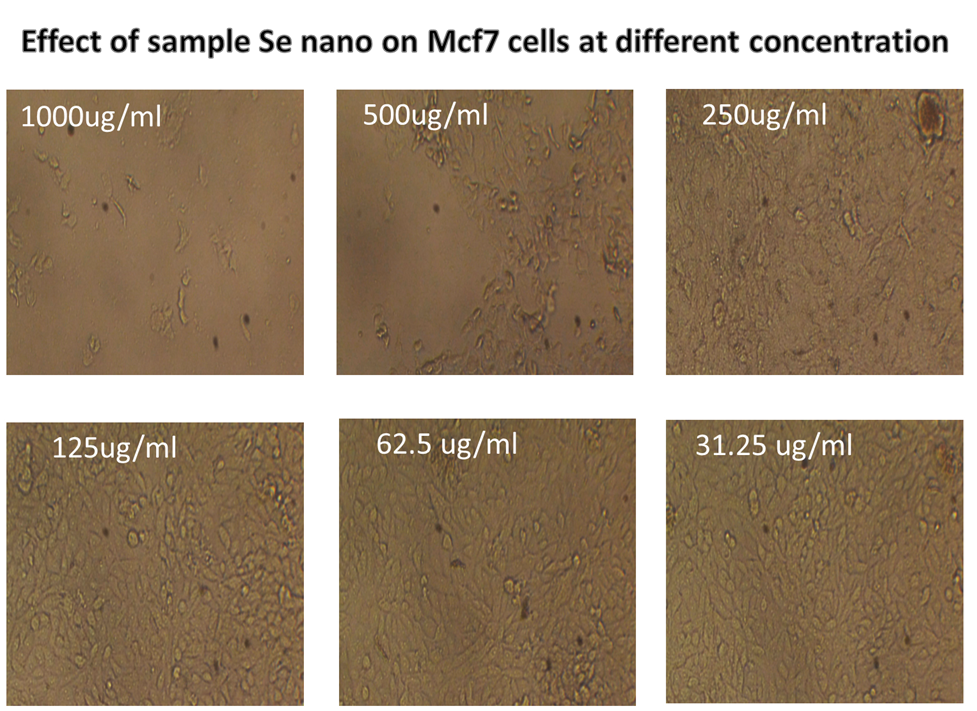 |
| 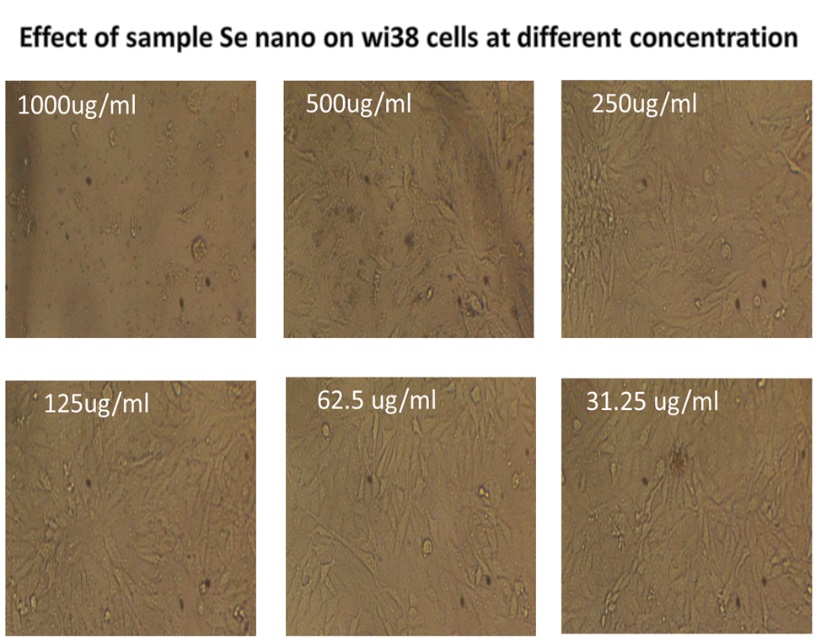 | 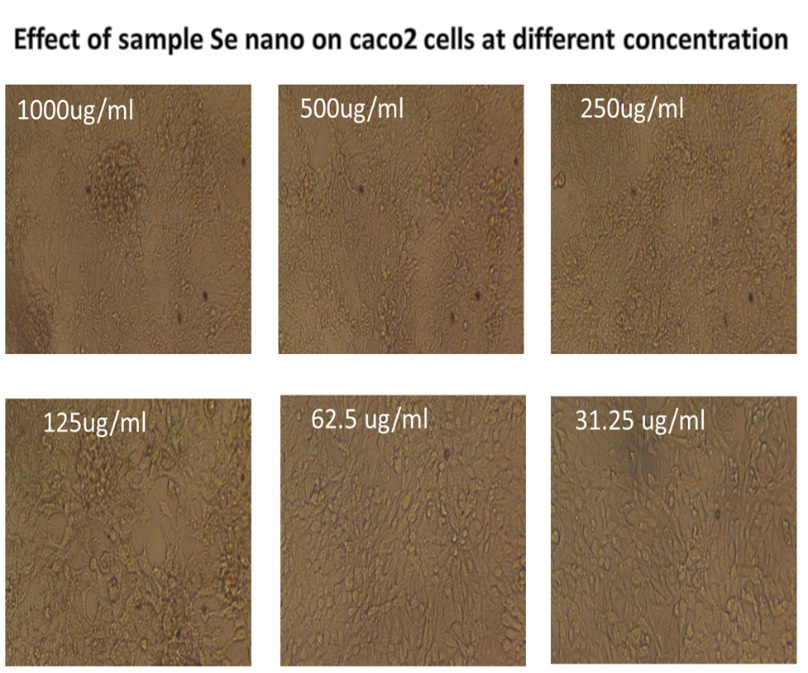 | 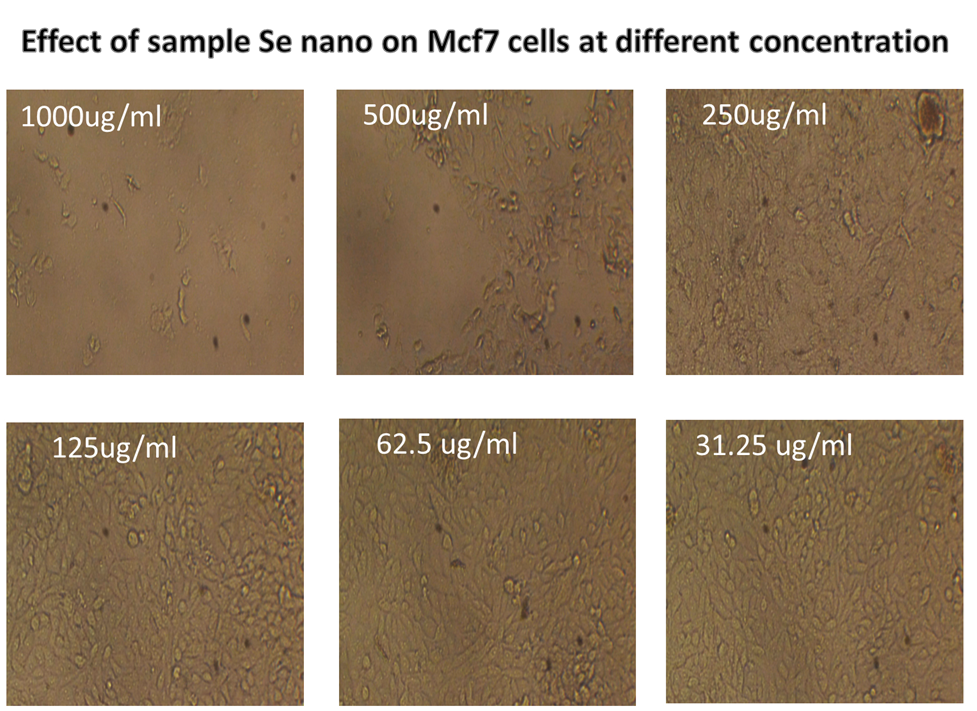 |
| 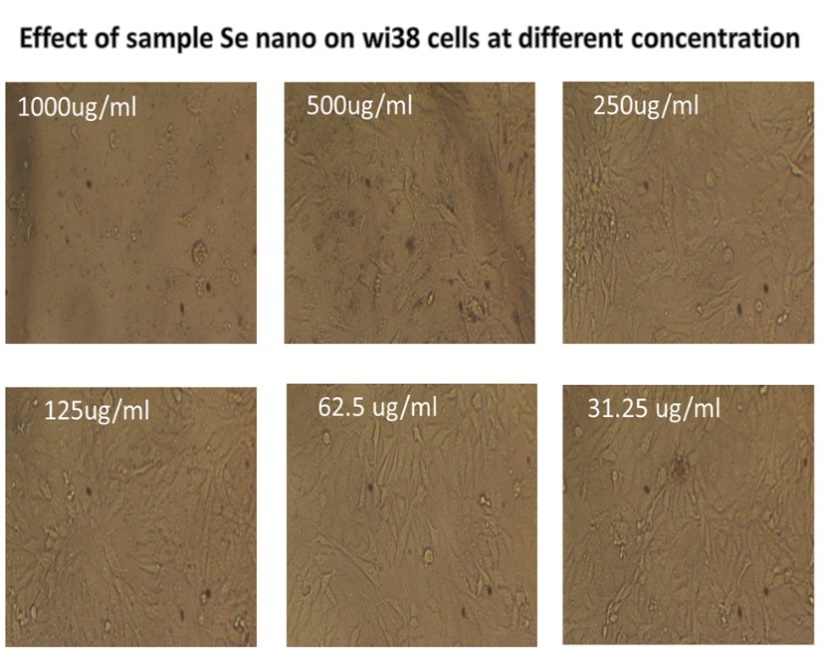 | 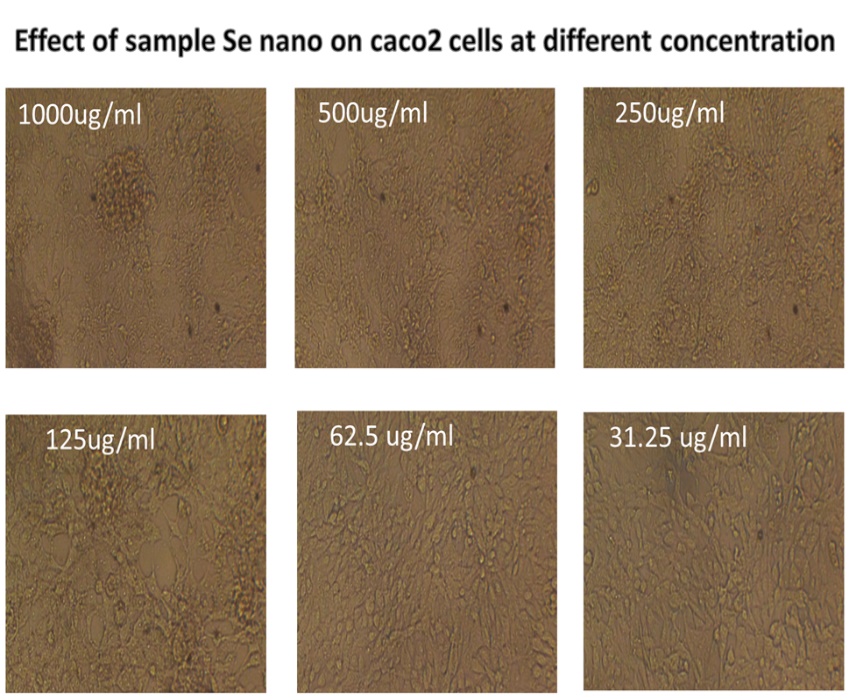 | 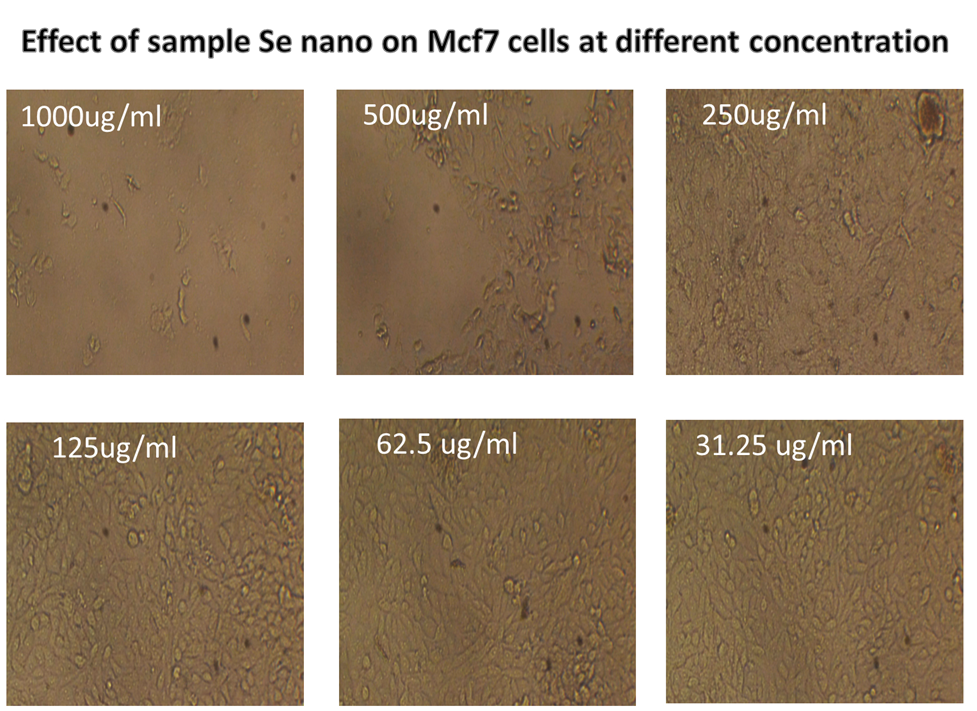 |
| 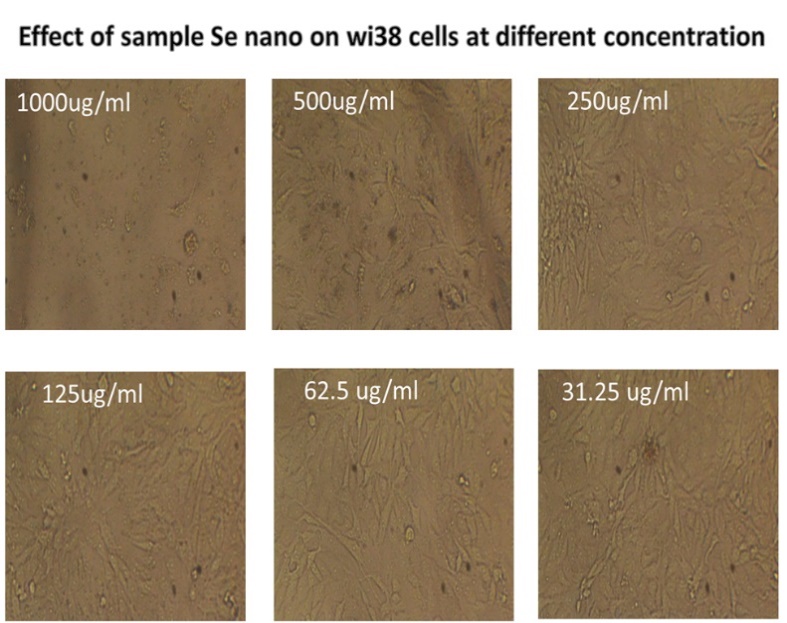 | 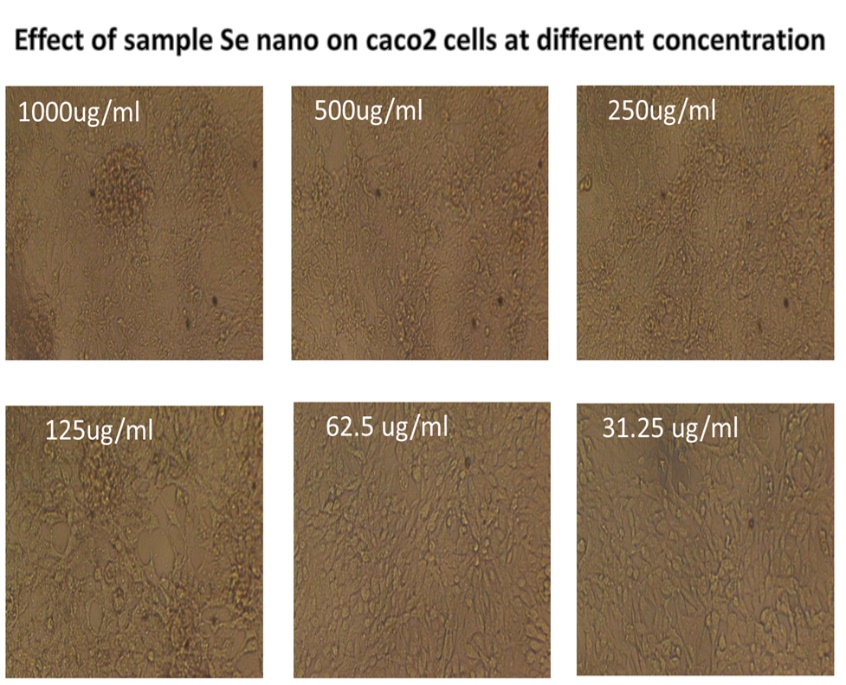 | 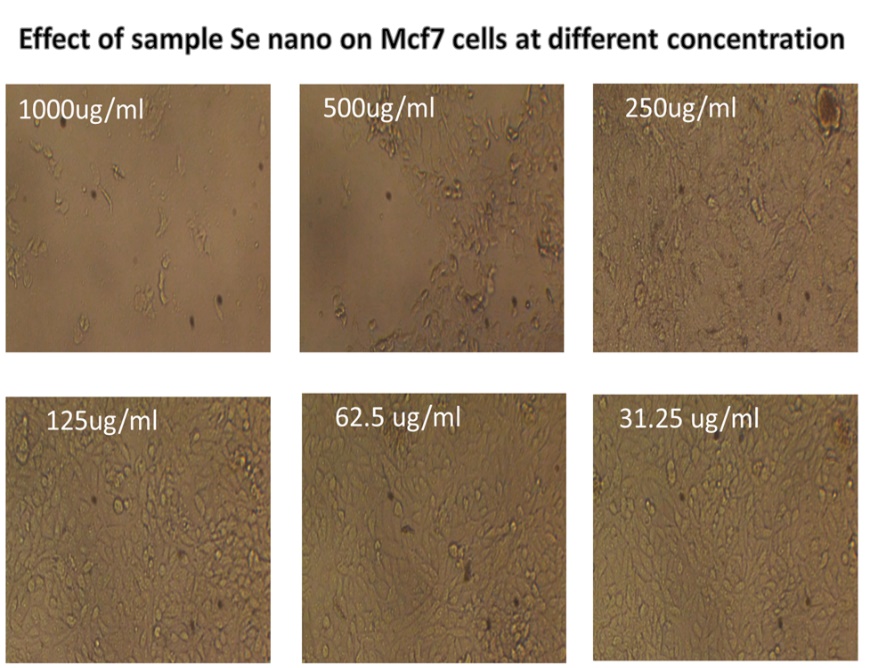 |
| 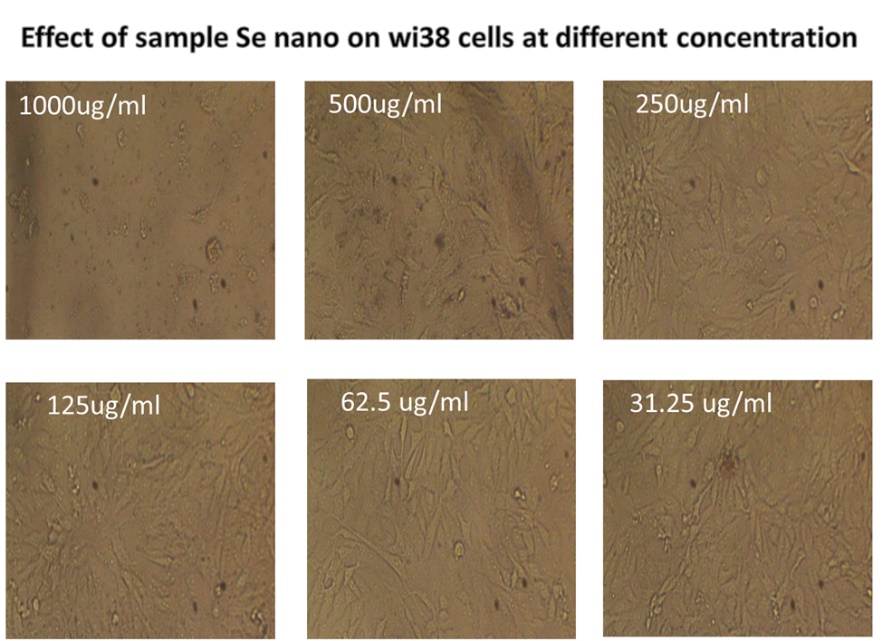 | 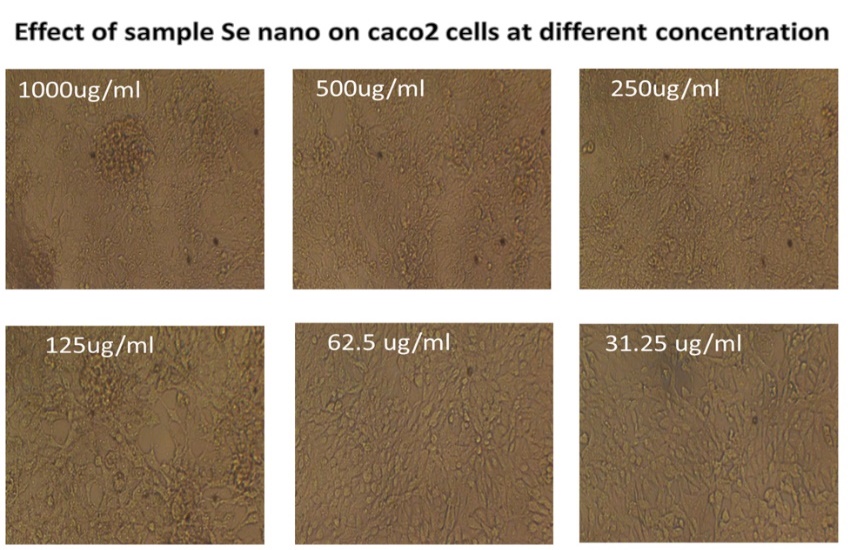 | 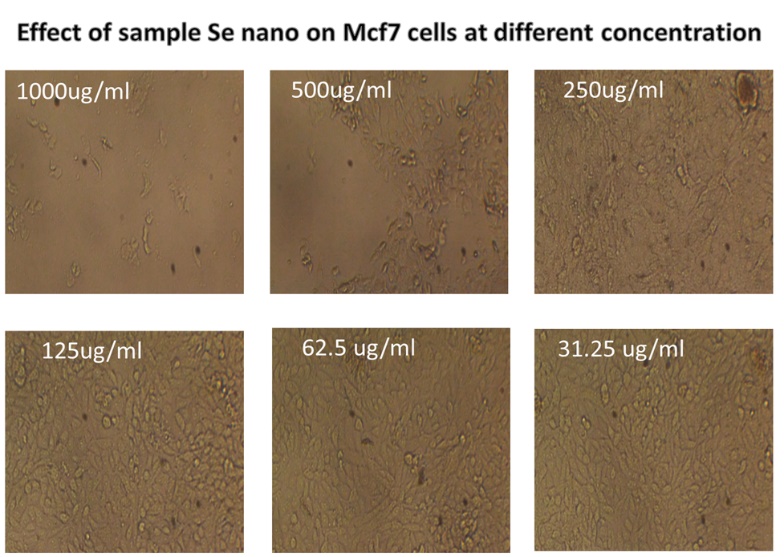 |

**Figure S3: Cytotoxicity effect of SeNPs different concentrations on MCF7, Caco2, and a normal cell, WI38.**
